# Dynamic forecasting module for chronic graft-versus-host disease progression based on a disease-associated subpopulation of B cells: a multicenter prospective study

**DOI:** 10.1016/j.ebiom.2025.105587

**Published:** 2025-02-12

**Authors:** Yuanchen Ma, Jieying Chen, Zhiping Fan, Jiahao Shi, Gang Li, Xiaobo Li, Tao Wang, Na Xu, Jialing Liu, Zhishan Li, Heshe Li, Xiaoran Zhang, Dongjun Lin, Wu Song, Qifa Liu, Weijun Huang, Xiaoyong Chen, Andy Peng Xiang

**Affiliations:** a Department of Gastrointestinal Surgery, The First Affiliated Hospital, Sun Yat-sen University, Guangzhou, 510080, China; b Center for Stem Cell Biology and Tissue Engineering, Key Laboratory for Stem Cells and Tissue Engineering, Ministry of Education, Sun Yat-Sen University, Guangzhou, Guangdong, 510080, China; c National-Local Joint Engineering Research Center for Stem Cells and Regenerative Medicine, Zhongshan School of Medicine, Sun Yat-Sen University, Guangzhou, Guangdong, 510080, China; d Department of Hematology, Nanfang Hospital, Southern Medical University, Guangzhou, 510515, China; e Core Facility of Center, Zhongshan School of Medicine, Sun Yat-Sen University, Guangzhou, 510275, China; f Department of Histoembryology and Cell Biology, Zhongshan School of Medicine, Sun Yat-Sen University, Guangzhou, Guangdong, 510080, China; g Department of Hematology, The Seventh Affiliated Hospital, Sun Yat-sen University, Shenzhen, 518107, China

**Keywords:** cGVHD, B cell, Dynamic monitor, Machine learning, Prospective study

## Abstract

**Background:**

Predicting chronic graft-versus-host disease (cGVHD) progression has been challenging due to its dynamic nature and the lack of reliable real-time monitoring tools, necessitating substantial investments of time and financial resources for effective management. Consequently, identifying appropriate immune cell subsets or molecules as prognostic or predictive biomarkers for cGVHD is essential.

**Methods:**

Building on the pivotal role of B-cell homeostasis in cGVHD progression, we integrated spectral flow cytometry with advanced machine learning algorithms to systematically analyze the relationship between B cells and cGVHD progression. Leveraging the identification of a distinct B-cell subpopulation, we developed cGPS (cGVHD Progress Score), a user-friendly tool based on marker distribution. To validate cGPS, we conducted both retrospective and prospective multi-center studies involving 91 patients (25 non-GVHD and 66 cGVHD cases).

**Findings:**

We identified a distinct B-cell subpopulation characterized by CD27^+^CD86^+^CD20^−^, which can precisely distinguish cGVHD. Leveraging this discovery, we developed cGPS. The retrospective study highlighted the predictive power of cGPS, achieving an impressive area under the curve (AUC) of 0.98 for identifying non-GVHD patients prone to cGVHD and 0.88 for predicting disease progression in cGVHD patients. Notably, the prospective study highlighted cGPS's effectiveness, as it accurately predicted all instances of cGVHD development or progression within an average of three-month observation window.

**Interpretation:**

These findings validate cGPS as a highly effective and dynamic B cell-based tool for monitoring cGVHD progression, offering a crucial solution for prognosis and prediction of treatment effectiveness. The multicenter approach applied to both retrospective and prospective studies strengthen the reliability and adaptability of our findings. We are confident that cGPS is a highly competitive tool with great potential for clinical application.

**Funding:**

This work was supported by grants from the 10.13039/501100012166National Key Research and Development Program of China, 10.13039/501100013290Stem Cell and Translational Research (2022YFA1105000, 2022YFA1104100); the 10.13039/501100001809National Natural Science Foundation of China (82430050, 32130046, 82270230, 81970109); 10.13039/501100015956Key Research and Development Program of Guangdong Province (2023B1111050006); 10.13039/501100021171Guangdong Basic and Applied Basic Research Foundation (2023B1515020119); Key Scientific and Technological Program of Guangzhou City (2023B01J1002); Pioneering talents project of Guangzhou Development Zone (2021-L029); the Shenzhen Science and Technology Program (KJZD20230923114504008).

**Research in context**

**Evidence before this study**

Chronic graft-versus-host disease (cGVHD) significantly impacts patients' quality of life, necessitating dynamic and real-time monitoring tools for disease prognosis and prediction. Existing markers lack robust evidence for reliably predicting cGVHD progression. B cells play a pivotal role in cGVHD pathogenesis, with certain subpopulations, such as CD27+ memory B cells, being linked to effective cGVHD therapy. These findings highlight the potential of specific B cell subsets as candidates for prognosing and predicting cGVHD progression.

**Added value of this study**

This study introduces a novel monitoring tool, cGPS, derived from the B cell subset characterized by CD27+CD86+CD20−, which can precisely distinguish cGVHD. In order to enhance the reliability, we conducted a multicenter, retrospective and prospective studies. cGPS has proven its ability of accurately distinguish cGVHD progression patients, including prognosing non-GVHD who might develop into cGVHD and predicting cGVHD who have disease progressed.

**Implications of all the available evidence**

The findings validate cGPS as an effective tool for prognosing and predicting cGVHD progression. It offers a valuable solution for early diagnosis and treatment assessment, addressing a critical unmet need in clinical practice and paving the way for improved patient outcomes.

## Introduction

Chronic graft-versus-host disease (cGVHD) poses a significant challenge following allogeneic hematopoietic stem cell transplantation (allo-HSCT), often leading to substantial morbidity and mortality.^1^^,^^2^ While corticosteroids serve as the primary first-line treatment,^3^ their efficacy is limited and prolonged use leads to significant toxicity. Consequently, 50–60% of patients necessitate secondary treatment within two years due to poor clinical outcomes.^4^ Some second-line treatments have increased the response among patients, but the limited treatment options and lack of real-time disease activity monitoring continue to hinder cGVHD treatment and outcomes.^5^

Preemptive and individualized therapy is pivotal in future cGVHD management.^4^^,^^6^^,^^7^ However, recognizing the earliest signs and symptoms of cGVHD remains challenging.^8^ Although the National Institutes of Health (NIH) consensus projects enhanced diagnostic accuracy and severity scoring, they failed to prognose imminent cGVHD onset, leaving some at-risk patients undetected.^9^ Current consensus criteria only allow clinicians to assess a patient's current state without predicting future responses or outcomes, resulting in delayed therapeutic strategies and reliance on a trial-and-error approach for subsequent treatments.^10^ Usually, the therapeutic response should be assessed at 8–12 weeks as it is reported that most therapies take some time to reach therapeutic peak.^11^ However, if progression occurs after 4 or more weeks of treatment, new options must be considered.^11^^,^^12^ Importantly, avoiding progression to severe cGVHD is critical, as mild cGVHD is associated with better disease control and reduced mortality. This underscores the need for developing real-time and objective indicators for cGVHD prediction and/or disease staging.

B cells play a substantiated role in cGVHD pathogenesis. Numerous studies have revealed that there are B cell homeostasis alterations in cGVHD,^13^^,^^14^ including the presence of characteristic autoreactive B cells, delayed reconstitution of naive B cells,^15^ and alterations of signaling pathway activities, such as increased B cell activating factor (BAFF) signaling and abnormally activated BCR signaling.^16^, ^17^, ^18^, ^19^ Depletion of B cells by rituximab or selective targeting of B cell signaling pathways can be effective therapeutic approaches in patients with cGVHD.^20^^,^^21^ Our previous studies revealed that the mesenchymal stem cell (MSC)-based improvement of cGVHD was accompanied by alteration of naïve, memory, and regulatory B cell subsets, modulation of the plasma BAFF level, and the expression of BAFF-R on B lymphocytes.^22^ Observations that B cell changes are closely related to cGVHD outcome suggested that a B cell-based strategy might be a useful approach for monitoring cGVHD activity.

As an integrative approach to build a cGVHD disease activity evaluation module, we herein used spectral flow cytometry and a machine-learning model to assess B cell alterations in a cGVHD patient. Importantly, we employed a multi-center approach involving retrospective and prospective studies to develop and validate B cell-based disease prognosis and prediction system for cGVHD.

## Methods

### Study design and participants

Cohort 1: 5 healthy donors and 5 cGVHD patients were studied to identify a cGVHD-related cell population.

Cohort 2: 17 healthy donors, 31 cGVHD patients, and 15 non-GVHD patients from The Seventh Affiliated Hospital of Sun Yat-sen University and Nanfang Hospital, Southern Medical University were included. This cohort confirmed that this B cell population was disease-related, identified the optimal marker combination, and developed the cGPS module for improved accessibility. It also used to assess the module's performance in disease prognosis and prediction through retrospective analysis.

Cohort 3: Comprised 32 healthy donors, 35 cGVHD patients, and 10 non-GVHD patients from The Seventh Affiliated Hospital of Sun Yat-sen University and Nanfang Hospital, Southern Medical University. Healthy donors helped confirm that this B cell population was disease-related. Cohort 3 validated the cGPS module's ability to prognose non-GVHD group members' disease development risk and predict disease progression (DP) in cGVHD patients.

### Patients characteristics

The cGVHD patient inclusion criteria were as follows: 1) aged 15–65 years; 2) gender unrestricted; 3) diagnosed with malignant hematologic disease, received allogeneic hematopoietic stem cell transplantation at least 3 months; and 4) diagnosed with cGVHD according to the NIH criteria of 2005 and 2014. The exclusion criteria were: 1) presence of residual underlying malignant hematologic disease or disease progression; 2) treatment with rituximab (anti-CD20mAb) or graft failure, or experience of life-threatening infection. cGVHD was diagnosed and graded at the time of sample collection. Patients who had received HSCT but had not developed cGVHD at the time of sample collection were defined as non-GVHD patients. Peripheral blood samples were collected from all patients and healthy donors, with their informed consent. The blood acquired date after transplantation of cGVHD and non-GVHD and cGVHD onset date after transplantation of cGVHD patients have been calculated and there is no significant difference between each group (Figure S6).

### Grading of cGVHD

Organ scoring was independently assessed by at least two clinical hematologists who from the independent study adjudication committee mentioned previously.^23^ Organ scoring was conducted according to the 2005 and 2014 NIH consensus criteria for cGVHD.^24^^,^^25^ The evaluated organs included skin, oral mucosa, eyes, gastrointestinal tract, liver, lung, joints, and genitalia. The severity of organ grading was: mild, 1–2 organs maximum 1 point; moderate: 3 or more organs 1 point, or at least one organ 2 points or lung 1 point; severe: 3 points for at least one organ, or 2 or 3 points for the lungs. Based on this rule, lung cGVHD was evaluated independently. If GVHD or non-GVHD adverse events could not be identified, biopsy procedures were recommended and a study adjudication committee comprising experts in transplantation reviewed the findings. Final adjudication of GVHD or non-GVHD adverse events occurred by vote.

### Patients’ observation strategy

For non-GVHD the observation lasts at least 3 months, once a non-GVHD developed into cGVHD, the observation would terminate. For cGVHD the observation lasts at least 3 months, once a patient reaches DP or CR the observation would terminate.

### Peripheral blood mononuclear cell (PBMC) isolation

The peripheral blood was diluted with 1 × PBS at a ratio of 1:1, and lymphocytes were isolated by Ficoll–Paque density gradient centrifugation. The obtained lymphocytes were washed with PBS to remove debris and platelets, and then resuspended with PBS for further phenotype staining, or resuspended with lymphocyte cryopreservation solution and then frozen and transferred to liquid nitrogen for storage.

### Antibodies and flow cytometry

PBMC from cGVHD patients, non-GVHD patients, or healthy donors were stained for B cell phenotyping with a 20-marker panel that could accurately distinguish B cell subsets and indicate their developmental stage and activation status (Table S8), or with a five-marker panel for detecting specific B cell subsets. Antibodies were detailed in Table S8. Phenotyping data were collected using an Aurora (Cytek, USA) and a CytoFLEX (Beckman Coulter, USA) and analyzed using the FlowJo and CytExpert software packages. Round robin test has been applied to flow cytometry and spectral flow cytometry, the result shows that the data generated from these two platforms was comparable (Table S9).

### Downstream analysis of flow cytometry data

The transformed data from FlowJo underwent analysis with Seurat, following the tutorial guidelines (https://satijalab.org/seurat/articles/pbmc3k_tutorial.html).^26^ Given that the data were previously normalized, quality control and normalization steps were omitted. Parameters were adjusted to accommodate the spectral flow cytometry data structure, specifying 20 markers per cell. Batch effect removal was performed for cohort 1 data using the ScaleData function with the vars.to.regress argument. We utilized random forest machine learning models to compare marker set accuracies for cohort 1. Within the Seurat framework, candidate markers representing a cGVHD-related cell population were selected. Using Python's built-in combination function, we obtained all possible marker combinations. For each combination, a random forest machine model was constructed using Sklearn (version 0.23.2).^27^ Data were split into training and testing sets using train_test_split, allocating 70% for training and 30% for testing.

### Model selection criteria

We selected the models that could accurately distinguish cGVHD patients (label 1) and healthy donors (label 0) in cohort 1. Function *rfc.score* was used to calculate the accuracy of the markers. Models with accuracy score >0.90 would be selected and made further validation using cohort 2. After cohort1 filtered, the qualified model's parameter was fixed and no further training was performed, they were directly used to validate cohort2 disease samples and healthy samples. Models with an area under curve (AUC) score >0.90^28^ and the fewest markers in combination (2 or 3) were selected. These models were considered to represent a cGVHD-related cell population with acceptable accuracy.

### ROC analysis

ROC analysis was performed using R packages pROC (version 1.16.2). The roc curve was first calculated using function roc and plot with pROC build-in function ggroc. The confidence interval for threshold, specificity, sensitivity, accuracy was calculated with a build-in function ci.coords, with parameter best.policy set to random.

### Statistics

Any two categories (e.g., HD versus cGVHD or disease recovered versus disease progressed in the cGVHD group) were compared using Fisher's exact test. Wilcoxon rank sum test was used in comparing the two samples. All statistical analyses were performed using R (version 4.0.5). Machine learning model selection was performed on the python platform (Python3.6). All codes generated for each analysis are available upon request.

### Ethics

This study was conducted in compliance with the Helsinki declaration. All procedures involving human subjects were approved by the Ethics Committee of Nanfang Hospital, Southern Medical University (Ethical approval No. NFEC-2020-147). All patients gave written informed consent to participate in the study.

### Role of funders

The funding sources did not have a role in study design, data collection, data analyses, interpretation, or writing of report. The sole responsibility for the content of this publication lies with the authors.

## Results

### Cohort characteristics and B cell phenotyping profile

**Fig. 1:**
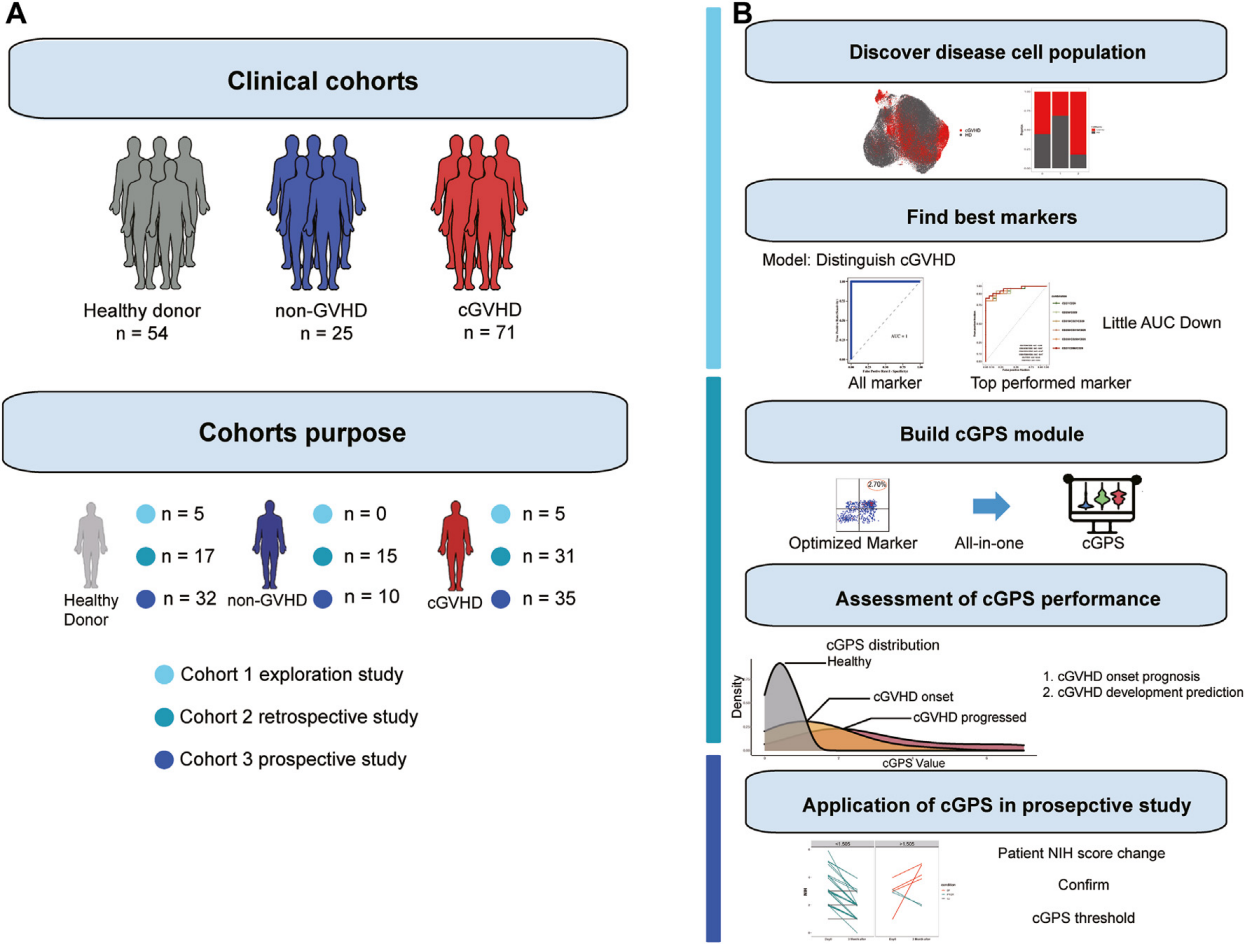
**Cohort design and the analysis process of this study**. (A) Patient included in each cohort study. (B) The analysis process of this study.

We recruited 150 participants from two independent medical institutes and categorized into three groups: 1) Control (n = 54), comprising healthy volunteers; 2) cGVHD (n = 71), diagnosed based on NIH criteria; and 3) non-GVHD (n = 25), individuals who received HSCT but lacked any apparent clinical feature of cGVHD as defined by the NIH criteria. These participants were divided into the three cohorts (Fig. 1A, Figure S3). In cohort1, five cGVHD patients and five healthy donors were enrolled for an exploratory study. In cohort2, 31 cGVHD patients, 15 non-GVHD patients, and 17 healthy donors were enrolled for a retrospective study. In cohort3, 35 cGVHD patients, 10 non-GVHD patients, and 32 healthy donors were enrolled for a further prospective study.

B cell phenotyping profiles of cGVHD patients, non-GVHD patients, and healthy controls were generated by spectral flow cytometry using a 20-marker panel that could accurately distinguish B cell subsets and indicate the developmental stage and activation status of B cells. The classical B cell subsets distinguishable by this phenotyping panel included transitional B cells, naïve B cells, memory B cells (including IgM^+^, marginal zone-like, and switched memory B cells), IgD^−^CD27^−^ double-negative B cells, and plasmablasts (Figure S1). As schematized in Fig. 1B, the B cell phenotyping profiles were further explored to find a cGVHD-related B cell subset, build a cGVHD disease activity evaluation module, and figure out the threshold for cGVHD management through retrospective and prospective studies. Clinical information on the cGVHD and non-GVHD group members are presented in Tables S1–S7.

### Identification of a cGVHD-related B cell subpopulation

**Fig. 2:**
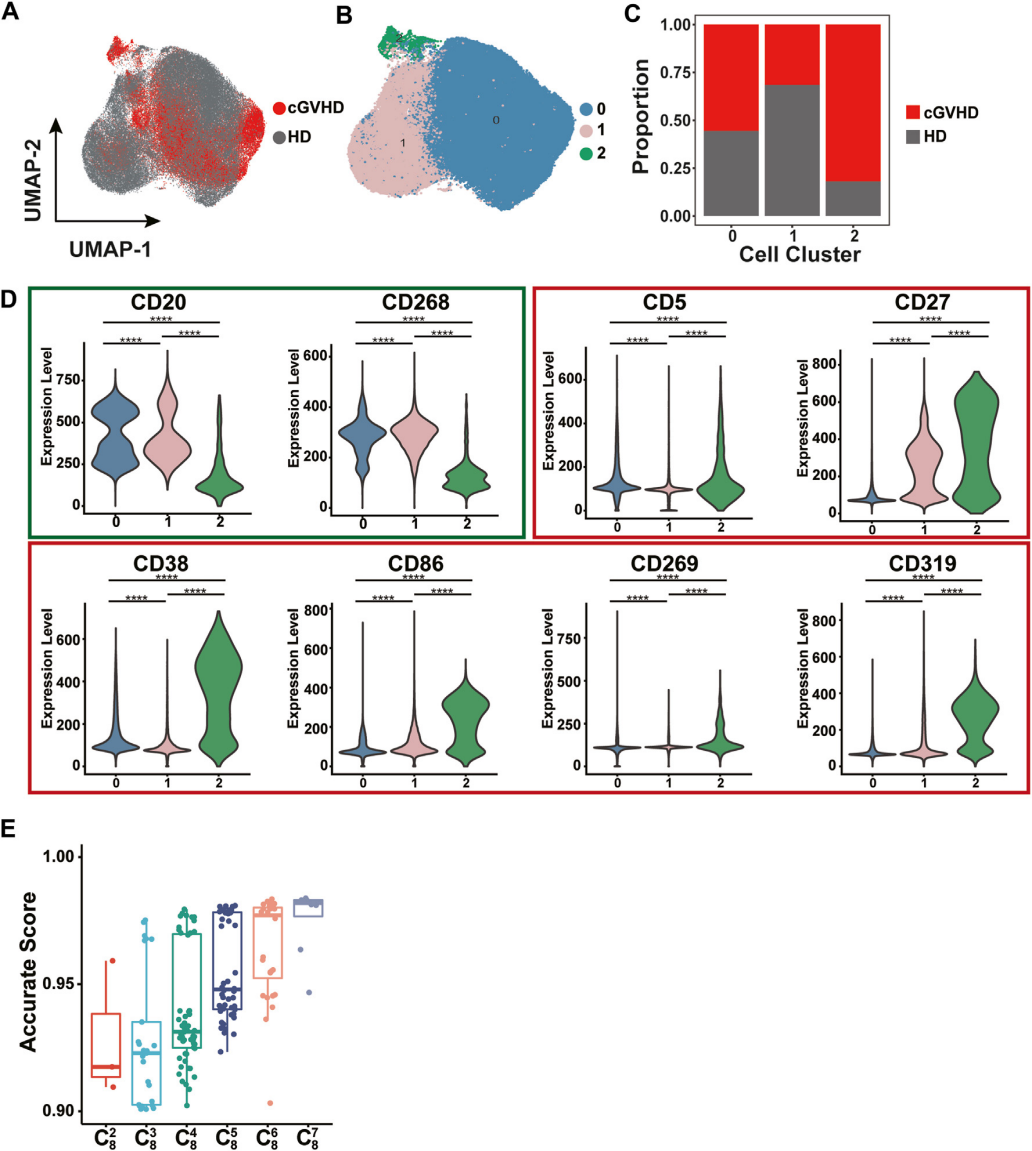
**cGVHD-related cell clusters and corresponding markers**. (A) UMAP plot for the B cell samples of 10 participants. Cell coloring indicates the sample source: red, cGVHD patients; gray, healthy donors. (B) UMAP plot for the B cell samples of 10 participants. Cells are colored to indicate participants category (left panel) and cell cluster (right panel). (C) Bar plot of cell proportions (y-axis) contained in each Louvain cell cluster (x-axis). Bar color indicates the sample source: red, cGVHD patients; gray, healthy donors. Bar length represents the percentage of the sample under the corresponding cluster. (D) Violin plot of the markers selected as potentially defining cGVHD-related cell clusters. The x-axis in each plot represents the Louvain cell clusters, while the y-axis represents the relative expression level of each marker. The width of each curve corresponds to the approximate frequency of data. The Wilcoxon rank sum test was applied to each comparison and the ∗∗∗∗ represents the significance *p* < 0.0001 (E) Box plot of machine learning model accuracy scores. For each marker combination, accuracy score above 0.90 was shown. The x-axis represents the marker combination category; for example, $C_{8}^{2}$ indicates a combination consisting of two non-repetitive markers randomly picked from all eight markers. The y-axis represents the machine learning model-calculated accuracy score for the ability of each combination to distinguish those with and without cGVHD. Each dot represents a machine learning model built from a distinct marker combination.

Spectral flow cytometry data obtained from cohort1 (10 participants; 5 cGVHD patients and 5 healthy donors) were used to identify a cGVHD-related B cell population. Uniform Manifold Approximation and Projection (UMAP) and Louvain clustering of cells^29^^,^^30^ yielded three cell clusters (Clusters 0–2; Fig. 2A and B). From the cell composition of each cluster, Cluster 2 was found to be a cGVHD-related cell cluster (Fig. 2C): cGVHD samples accounted for 82.0% of the Cluster 2 cells (1748 cells), while healthy donor samples accounted for only 18% (383 cells). To phenotypically characterize the cluster 2, we examined the cell surface phenotypes of each cluster. From among the 20 studied markers, CD20, CD268, CD5, CD27, CD38, CD86, CD269, and CD319 were identified as specific markers for Cluster 2 based on their expression levels (Fig. 2D). We also compare non-GVHD and cGVHD samples and the similar cluster could still be detected (Figure S4).

We used these eight markers to generate an unbiased random forest machine learning model and found that these markers could accurately label a disease-associated cell cluster. We then explored whether these eight markers could be further streamlined, with the goal of improving the clinical applicability of our method. We iterated all possible marker combinations and evaluated their accuracy. As presented in Fig. 2E, the accuracy scores indicated that certain combinations of only two or three markers could substitute for the full eight-marker panel with little loss of accuracy.

### Optimal marker combination for identifying a cGVHD-related B cell subpopulation

**Fig. 3:**
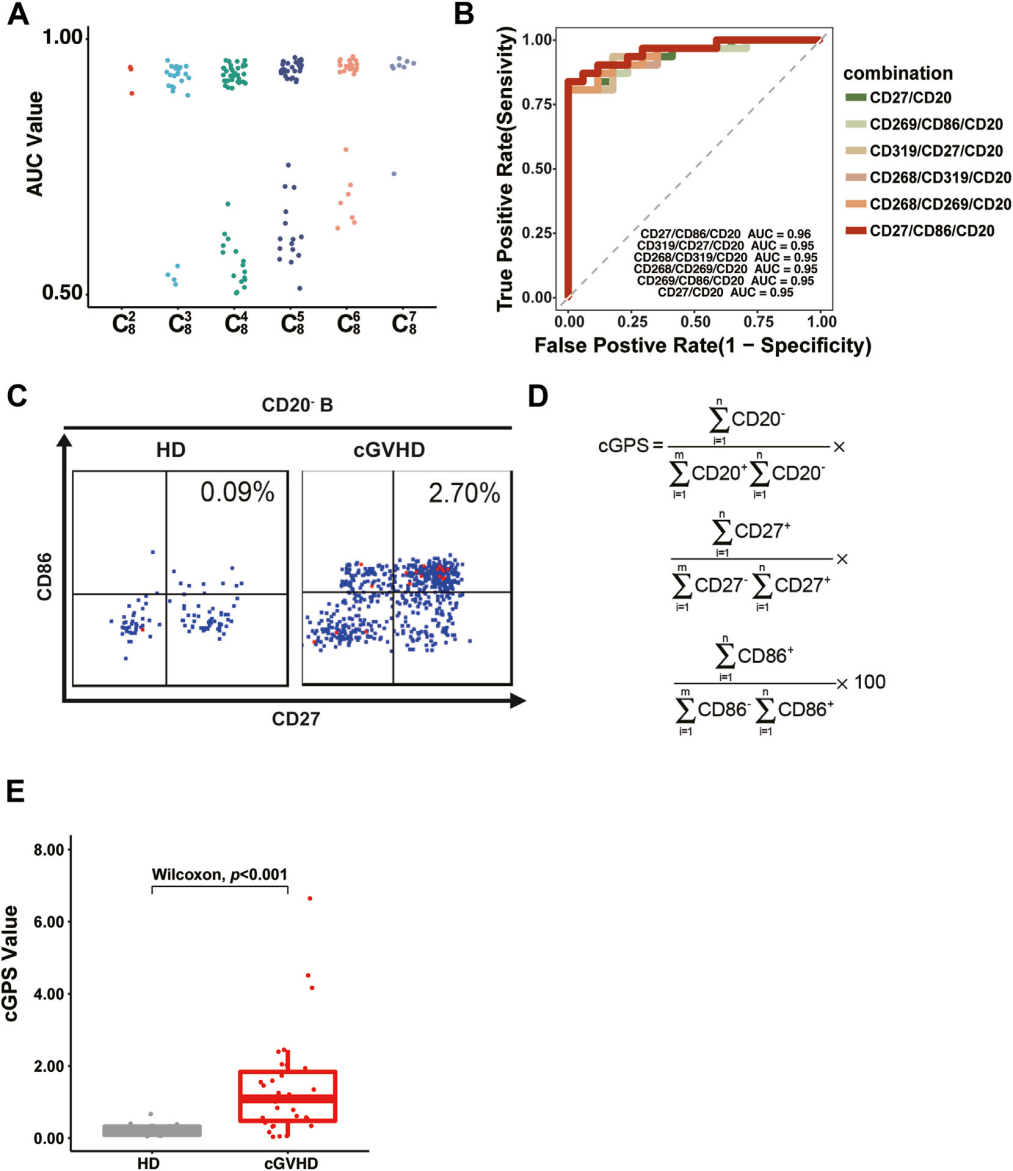
**Optimization of marker combinations and building of the cGPS**. (A) Scatter plot showing the results obtained using each high-performing model on cohort 2. The x-axis presents the marker combinations; for example, $C_{8}^{2}$ indicates a combination consisting of two non-repetitive markers randomly picked from all eight markers. The y-axis presents the corresponding AUC scores. (B) AUC plot showing the performance of the best six-marker combination models. The x-axis presents the false-positive rate and the y-axis presents the true-positive rate. The color of each curve indicates the marker combination. (C) The representative flow cytometry plot showing the CD20^−^CD27^+^CD86^+^ cell proportions among cGVHD and healthy donor samples. The x axis represents CD27 cell and y axis represents CD86 cells, the digital number on the top right square indicate the CD27 and CD86 double positive cell percentage. (D) Formula used to transfer the results from a marker combination into a more accessible scoring system. (E) Statistical analysis of differences in the cGPS among cGVHD and healthy donor samples. The x-axis represents the healthy donors (HD, gray) and cGVHD patients (red). The y-axis presents the corresponding cGPS. The Wilcoxon represents Wilcoxon rank sum test.

To identify a simple marker combination that could identify a cGVHD-related B cell subset, we used an independent cohort (48 participants; 31cGVHD patients from two independent medical institutes and 17 healthy donors) and marker combinations with accuracy scores >0.90 (20 combinations; Fig. 3A). The three-marker combination of CD20/CD27/CD86 yielded the best AUC score (AUC score = 0.96, Fig. 3B) emerging as the optimal option.

Flow cytometry further confirmed that the CD20/CD27/CD86 marker set effectively identified a cGVHD-related B cell subpopulation, with CD27^+^CD86^+^CD20^−^ B cells being highly prevalent in cGVHD patients and nearly absent in healthy donors (Fig. 3C). To enhance the clinical utility of this marker set, we leveraged the distribution of CD27^+^CD86^+^CD20− cells to develop a score known as cGPS (cGVHD Progress Score). This score ranged from 0 to 100 succinctly summarizes the frequency of CD27^+^CD86^+^CD20− B cells, providing a direct reflection of the cGVHD-associated B cell subsets. Initially, the module calculates the CD27^+^CD86^+^CD20− cell frequencies and employs a formula outlined in Fig. 3D to translate these frequencies into a single, easily interpretable score. This score captures the disparity between cGVHD pathology and a healthy state, yielding a personalized cGPS value for each participant within the cohort (*p* = 3.00 × 10^−5^, Fig. 3E).

### Using a cGPS threshold to predict progression risk among non-GVHD patients

Prognosing the imminent onset of cGVHD at an early stage remains challenging, as non-GVHD patients do not exhibit obvious signs per NIH consensus criteria during the early stages of cGVHD development.^31^, ^32^, ^33^ Hence, we tested whether cGPS could potentially prognose progression of non-GVHD patients to cGVHD. Details on the non-GVHD patients enrolled into Cohort-2 are presented in Table S2. According to their progression (or lack thereof) to cGVHD within an average of 3-month period observation, the subjects were categorized as stable state (those remaining in non-GVHD status) and active state (patients that developed cGVHD). The patients of the two groups had similar baseline clinical characteristics (Table S2).

**Fig. 4:**
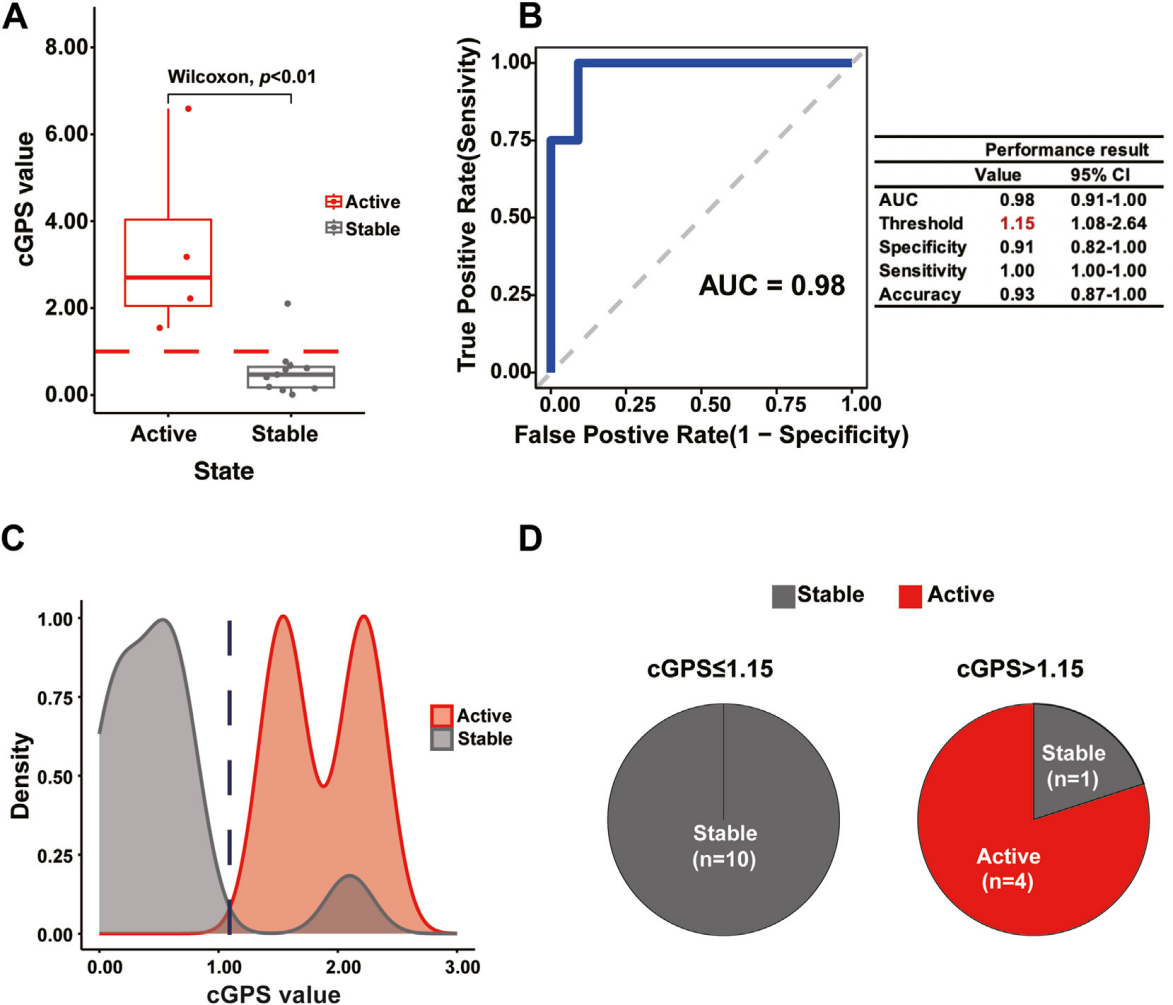
**Retrospective study of cGPS in non-GVHD patients**. (A) Statistical analysis of cGPS among patients with stable state (those remaining in non-GVHD status) and active state (patients that developed cGVHD). The x-axis presents the two non-GVHD groups. The y-axis presents the cGPS. The red dashed line represents 1.00, indicates the approximate cGPS threshold for separating the two groups. The Wilcoxon represents Wilcoxon rank sum test. ∗∗: *p* < 0.01. (B) ROC analysis identifying the threshold of cGPS that can be used to separate active group on non-GVHD patients. The left panel shows the AUC plot, where the x-axis presents the false-positive rate and the y-axis presents the true-positive rate. The right panel shows various parameters, including the AUC, threshold, specificity, sensitivity, and accuracy values, with 95% confidence intervals (CI). (C) Density plot of the two non-GVHD groups. The x-axis presents the cGPS, while the y-axis presents the frequency of each score. The area color indicates the group: gray, Stable groups; and red, Activate groups. The dark blue dashed line represents the threshold cGPS of 1.15. (D) Pie plot of cGPS distribution by group: The left pie plot represents cGPS ≤1.15 and the right pie plot represents cGPS >1.15; gray indicates patients of the stable group and red indicates those of the active group.

Statistical analysis revealed significant differences in cGPS values between stable and active groups (*p* < 0.01). Patients in the stable group predominantly had cGPS <1.00, while those in the active group had cGPS >1.00 (Fig. 4A), suggesting a potential threshold around 1.00 to distinguish between stable and active states. Our receiver operating characteristics (ROC) analysis revealed that the precise cGPS threshold Youden index for this cohort was 1.15; indeed, 100% of non-GVHD patients scoring below this threshold were in stable state (Fig. 4B). In addition to being able to distinguish the state of non-GVHD patients (Fig. 4C), this cGPS threshold could also be used to prognose whether non-GVHD patients would develop into cGVHD: As shown in Fig. 4D, four out of five (80%) non-GVHD patients with cGPS >1.15 developed into cGVHD during the evaluation period, whereas all 10 non-GVHD patients with cGPS ≤1.15 remained in stable state.

### Using a cGPS threshold to estimate the treatment efficiency of cGVHD

Predicting the likelihood of disease progression in cGVHD patients quickly and efficiently poses a challenge.^34^^,^^35^ As NIH consensus criteria for organ scoring was a follow-up evaluation system, cGVHD real time state monitor was hard to captured. Since the cGPS module was generated from the disease-related B cell subset, we questioned whether it might be able to distinguish disease-progressed cGVHD patients. To address this question, we classified the cGVHD patients of Cohort-2 as having stable disease (SD), partial response (PR), complete response (CR), or disease progression (DP), and collected them into two groups: the DP group and the non-DP group (PR/CR/SD groups). The baseline clinical characteristics of the two groups are shown in Table S3.

**Fig. 5:**
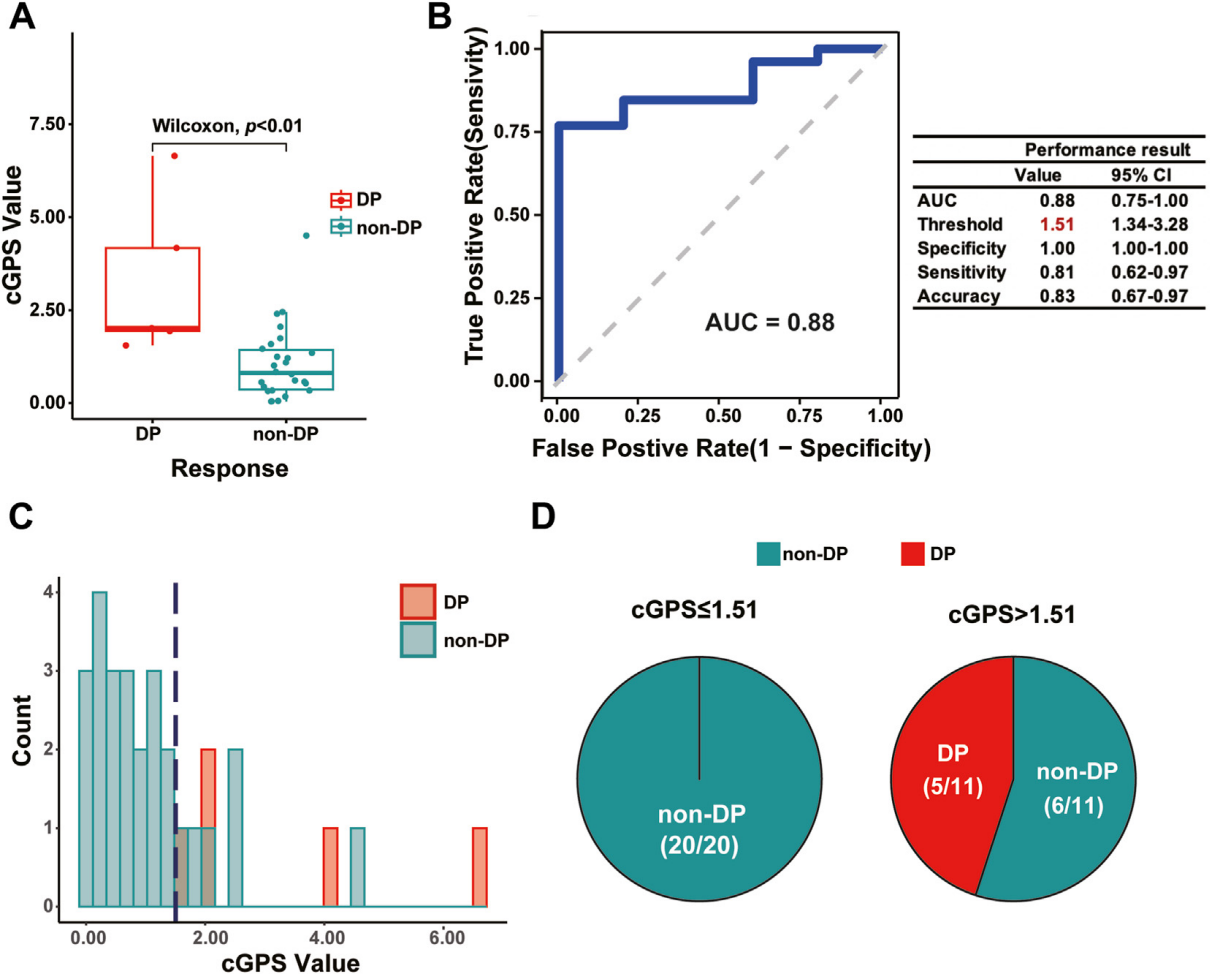
**Retrospective study of cGPS among additional cGVHD patients**. (A) Statistical analysis of cGPS between the two cGVHD groups: DP (disease progression) and non-DP (PR: partial response, CR: complete response, SD: stable disease). ∗∗∗: *p* < 0.001. The x-axis presents the two cGVHD groups, while the y-axis presents the cGPS score. The red dashed line indicates the approximate cGPS threshold separating the two cGVHD groups. The Wilcoxon represents Wilcoxon rank sum test. (B) ROC analysis identifying the cGPS threshold that can be used to separate out the DP group of cGVHD patients. The left panel represents the AUC plot; the x-axis presents the false-positive rate and the y-axis presents true-positive rate. The right panel presents various parameters, including the AUC, threshold, specificity, sensitivity, and accuracy values, with 95% CI. (C) Histogram plot for the two cGVHD groups. The x-axis presents the cGPS, while the y-axis presents the patient number of each score. The bar color indicates the group: green, non-DP (PR/CR/SD) groups; and red, DP groups. The blue dashed line indicates the threshold cGPS of 1.51. (D) Pie plot of cGPS distribution by category: The left pie plot presents cGPS ≤1.51 and the right pie plot presents cGPS >1.51; green, non-DP (PR/CR/SD) cGVHD patients; and red, DP cGVHD patients.

Significant differences in cGPS were observed between the DP and non-DP groups (*p* < 0.01), with a discernible threshold around 1.50 (Fig. 5A). ROC analysis indicated that a cGPS of 1.51 effectively separated the DP and non-DP groups (Fig. 5B). Further exploration of cGPS distribution between the two groups validated this finding, showing clear separation using a cGPS threshold of 1.51 (Fig. 5C). Notably, no patient with cGPS ≤1.51 progressed to the DP state, while 45.50% of patients with cGPS >1.51 entered the DP state (Fig. 5D).

### Using the above-identified cGPS thresholds in a prospective study

**Fig. 6:**
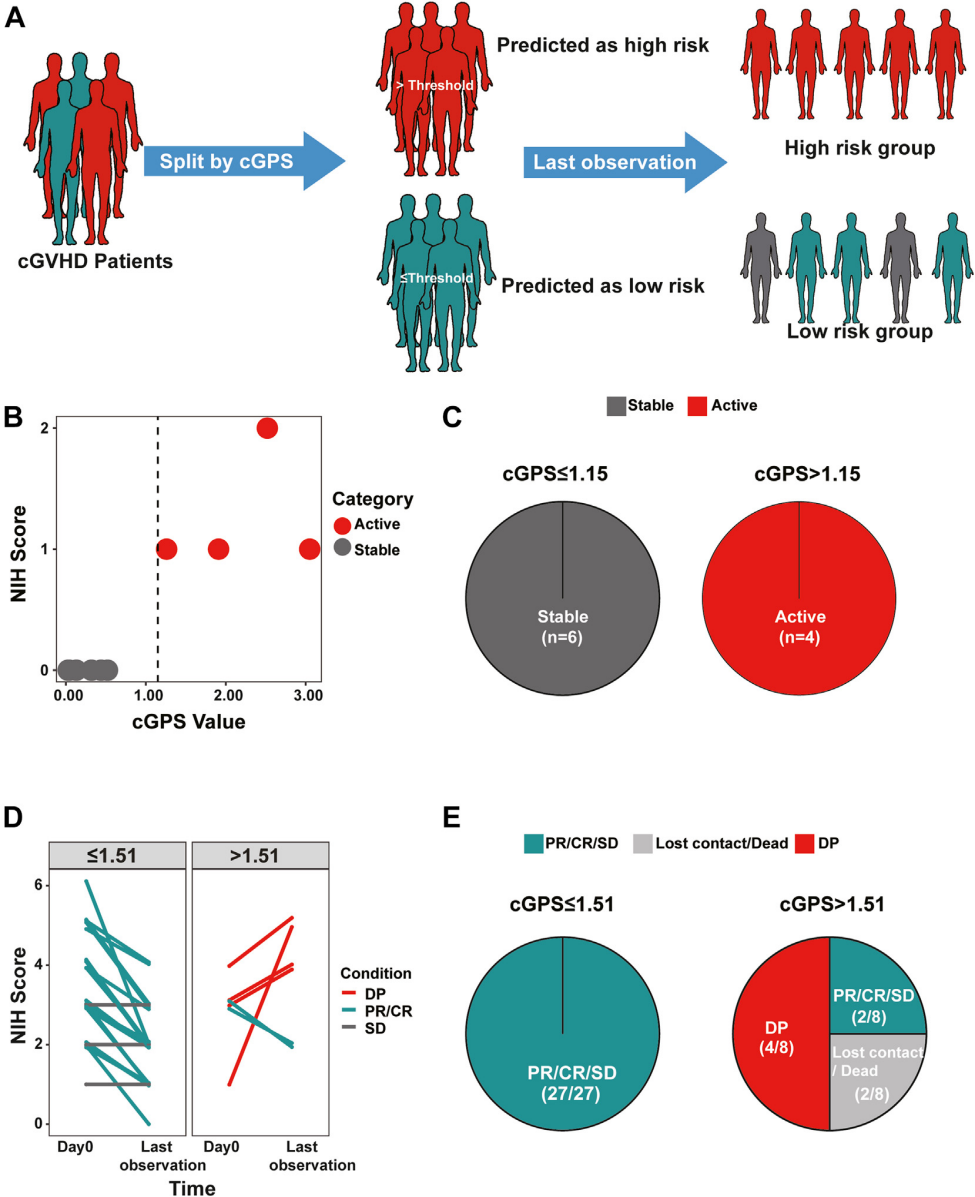
**Prospective study of cGPS among cGVHD and non-GVHD patients**. (A) The workflow of the prospective study. cGVHD patients were separated into high- and low-risk groups based on their cGPS. Then till the last observation, the states of these patients were re-evaluated based on the NIH consensus criteria, and the accuracy of the cGVHD classification was assessed. (B) Scatter plot of non-GVHD patients last observation and the first evaluation. The x-axis presents the cGPS, the y-axis presents the NIH consensus score, the dot color indicates whether the patient had developed cGVHD (red) or remained non-GVHD (gray), and the black dashed line represents the cGPS threshold at 1.15. C) Pie plot of cGPS distribution: The left pie plot presents the low-risk ground (cGPS ≤1.15)and the right pie plot represents high-risk ground (cGPS >1.15); gray indicates stable patients and red indicates active patients. (D) Segment plot of differences in NIH scores between the first evaluation (Day 0) and last observation, among patients categorized based on initial cGPS ≤ or >1.51. The x-axis presents the time point and the y-axis presents the NIH score; red indicates disease progression (DP), green indicates partial or complete response (PR/CR), and gray indicates stable disease (SD). (E) Pie plot of cGPS distribution: The left pie plot presents low-risk groups (cGPS ≤1.51)and the right pie plot represents high-risk groups (cGPS >1.51); green indicates PR/CR/SD cGVHD patients and red indicates DP patients, and gray indicates lost contact or dead.

To validate the potential utility of cGPS thresholds for cGVHD management, we conducted a prospective study in a new cohort. 77 participants from 2 independent medical institute (Cohort 3: 35 cGVHD patients, 10 non-GVHD patients, and 32 healthy donors). Each participant's cGVHD-related B cell subpopulation was analyzed, cGPS values were generated (Figure S2). The cGPS values between healthy donors and cGVHD patients in cohort3 remain significant different (Figure S5). Using cGPS thresholds, cGVHD and non-GVHD patients were categorized into high-risk and low-risk groups. Within an average of 3-month observation window, patients were re-evaluated with NIH consensus criteria and compared to our patient classification. The whole process was summarized in Fig. 6A. Clinical information is summarized in Tables S4, S5. Results for comparing non-GVHD patients are shown in Fig. 6B and C. Remarkably, all non-GVHD patients in the high-risk group (initial cGPS >1.15) were diagnosed with cGVHD at the second evaluation, while those in the low-risk group (initial cGPS ≤1.15) remained stable, achieving 100% prognostic accuracy for non-GVHD patients who would become disease-progressed.

We then assessed the ability of cGPS to predict the disease progression of cGVHD. As shown in Fig. 6D and E, all 24 patients in the low-risk group (with an initial cGPS ≤1.51) were diagnosed as PR/CR/SD (i.e., non-DP) at the second evaluation. The accuracy of the cGPS module was thus 100% for predicting no disease progression. Of the eight patients in the high-risk group (with cGPS >1.51), four (50.00%) were classified into the DP group at the second assessment, one was lost to follow-up, and one died of infection. The results of our prospective study therefore collectively indicated that the cGPS module may be a powerful tool for predicting the future course of disease for both cGVHD and non-GVHD patients in the clinic. This strategy should help doctors obtain timely and effective information that can be leveraged to improve patient care.

### Overall summary

We herein leveraged previous reports that aberrant B-cell homeostasis could influence the progression of cGVHD to develop a new strategy for recognizing and predicting the disease. Using spectral flow cytometry in a small cohort, we successfully identified a cGVHD-related subpopulation of B cells and delineated the defining markers of this subgroup. By applying machine learning in an independent cohort, we derived a simple marker combination and used it to develop an all-in-one scoring module named cGPS. ROC analysis was used to identify cGPS thresholds that can be used to evaluate disease progression in cGVHD and non-GVHD patients of this cohort. Finally, we performed a multicenter prospective study in a new cohort using these thresholds, and verified that our cGPS module could be a powerful tool for predicting the future course of disease among cGVHD and prognosing the development of non-GVHD patients in the clinic.

## Discussion

In this study, we identified a cGVHD-related B cell subpopulation using spectral flow cytometry, this identified cGVHD-related B cell subpopulation expressed the surface markers CD19^+^CD20^−^CD27^+^CD38^+^IgD^−^. Moreover, CD269 and CD319 were highly expressed, suggesting that the cGVHD-related B cell subpopulation could correspond to plasmablast-like cells. Plasmablasts are generally expected to play an important role in the etiology of cGVHD.^2^ Increased levels of B cell activation factor (BAFF) disrupt the negative selection mechanisms to prime the survival of autoreactive B cells, which leads to altered peripheral B cell compartment including post-germinal center plasmablasts (CD27^+^CD38^hi^IgD^–^).^15^ These abnormal plasmablasts have pathologic functions, including autoantibody production, cross-presentation to T cells, and B cell cytokine production, all of which could promote the development of cGVHD.^36^

Moreover, existing research has shown that changes in B cell phenotypes are closely linked to the progression of GVHD. In acute GVHD (aGVHD) patients, there is a marked upregulation in the expression of CD86 (an activation marker) and CD20 (a marker of mature B cells), which mirrors the changes seen in B cells during infections.^37^ This alteration reflects the heightened immune activation characteristic of aGVHD. However, steroids and calcineurin inhibitors (CNI) can partially reverse these changes.^38^ Furthermore, CD27+ memory B cells are often depleted in aGVHD, which may impair long-term immunity in patients who are already immunocompromised from prior treatments.^39^ This depletion of memory B cells increases the risk of future infections and compromises the effectiveness of the immune response over time.^40^

We further found that the levels of these aberrant B cells were positively associated with disease progression. This is consistent with recent reports that there is a strong correlation between the development of cGVHD and high levels of plasmablasts^41^^,^^42^ and that an increase of plasmablasts accompanies cGVHD progression.^43^ Given the above, we believed that our findings were rational from an etiological perspective.

Previous studies have identified several markers for cGVHD prognosis, including cytokines,^44^ T or B cell subpopulations,^45^^,^^46^ or body fluids.^47^ These markers have demonstrated efficacy in cGVHD prognosis. However, none of them have achieved AUC above 0.90. Our cGPS AUC have reached 0.98 exhibited more competitive results, achieving optimal sensitivity and specificity, highlighting its promising clinical applications. Additionally, we observed that cGPS remained consistent over time; some non-GVHD patients in our study were followed for 6 months, and those with cGPS below the threshold maintained a non-GVHD state. These findings suggest that our module can offer prolonged and sensitive warnings of disease progression.

On the other hands, our cGPS module showed a great capacity for reflecting treatment efficacy in cGVHD patients: 100% patients with cGPS ≤1.51 showed complete or partial responses to treatment. This finding underscores the potential of cGPS to guide clinicians in optimizing treatment strategies, especially for patients with mild but persistent symptoms, as mild cGVHD is linked to better disease control and lower mortality.

Among the patients with cGPS >1.51, the treatment was adjusted in four cases, with the addition of a therapy such as MSC transplantation. Upon the treatment adjustment, the cGPS of these patients decreased to ≤1.51 and their disease improved, as found in a follow-up observation (data not shown). In real situation, some cGVHD may not have enough circulating B cells.^48^ After checking all participants in our studies we found that each cGVHD patients have CD19^+^CD20^−^ B cells above 1%. Thus, more studies were needed to discover how cGPS could help those cGVHD patients whose circulating B cell numbers was insufficient.

Although numerous markers have been identified for cGVHD prognosis or prediction, few studies have included prospective research, and some markers are organ-specific, limiting their clinical application.^49^, ^50^, ^51^ Here, we included a multicenter prospective study to increase our sample size and improve the strength of our conclusions. We rigorously examined all participant information to avoid selective bias. Notably, two patients from a third medical institute in our prospective study—one with a cGPS below 1.51 and the other above 1.51—showed consistent clinical responses after an average of three-month check. This finding reinforces the reliability and adaptability of cGPS for monitoring cGVHD development. For sample size calculations, we set the power to 0.8, the effect size to 0.5, and the significance level to 0.05, resulting in a required sample size of >33 to minimize type I and type II errors. Our prospective study included 35 cGVHD patients, meeting this requirement and ensuring that cGPS 1.51 is a reliable threshold for evaluating cGVHD treatment efficacy. However, we only recruited 10 patients to study the ability of cGPS to provide early progression warnings for cGVHD, yielding an approximate power of 0.30. Additionally, we observed differences between cGVHD and non-GVHD patients regarding the day of blood sampling post-transplantation and the use of immunosuppressive medications. Since the quality of cell reconstruction depends on these factors, third party validation was needed to optimize our prediction and prognosis thresholds. While the limited sample size hinders strong conclusions about using cGPS 1.15 as a threshold in the non-GVHD group, the combination of retrospective and prospective study designs, along with the significant effect observed, suggests this threshold can predict the risk of a non-GVHD patient developing cGVHD. Nonetheless, cGPS 1.15 might be an approximation, and further research is needed to refine this threshold.

### Conclusion

By combining the power of spectral flow cytometry and machine learning, we successfully invented a B cell-based dynamic forecasting module, named cGPS, for chronic graft-versus-host disease. This module may enable sensitive and dynamic monitoring of cGVHD progress and thus may enable to prognose non-GVHD. Our findings cater to the clinical needs of cGVHD patients, and we believe that the cGPS module has the potential to become an ideal tool for estimating the progression of cGVHD.

## Contributors

APX, XC, YM and JC conceptualized and designed the project; APX, XC and WS supervised the research; YM, JC, JS, GL, XL, TW, JL, ZL, HL and XZ performed experiments and/or data analysis; DL, QL, ZF and NX contributed to the provision of the patients and the collection of data. JC, JS, XL and XC performed the spectral flow cytometry data analysis. YM and WH performed the bioinformatic analysis. MY, XC, JC and WH drafted the manuscript. APX, XC and MY revised the manuscript. All authors read and approved the final manuscript.

## Data sharing statement

The datasets used and/or analyzed during the current study are available from the corresponding author on reasonable request.

## Declaration of interests

All authors declare they have no conflicting interest.

## References

[1] D'Souza A., Fretham C., Lee S.J. (2020). Current use of and trends in hematopoietic cell transplantation in the United States. Biol Blood Marrow Transplant.

[2] MacDonald K.P.A., Hill G.R., Blazar B.R. (2017). Chronic graft-versus-host disease: biological insights from preclinical and clinical studies. Blood.

[3] Sullivan K.M., Witherspoon R.P., Storb R. (1988). Prednisone and azathioprine compared with prednisone and placebo for treatment of chronic graft-v-host disease: prognostic influence of prolonged thrombocytopenia after allogeneic marrow transplantation. Blood.

[4] Flowers M.E., Martin P.J. (2015). How we treat chronic graft-versus-host disease. Blood.

[5] Penack O., Marchetti M., Ruutu T. (2020). Prophylaxis and management of graft versus host disease after stem-cell transplantation for haematological malignancies: updated consensus recommendations of the European Society for Blood and Marrow Transplantation. Lancet Haematol.

[6] Wolff D., Hilgendorf I., Wagner-Drouet E. (2019). Changes in immunosuppressive treatment of chronic graft-versus-host disease: comparison of 2 surveys within allogeneic hematopoietic stem cell transplant centers in Germany, Austria, and Switzerland. Biol Blood Marrow Transplant.

[7] Saidu N.E.B., Bonini C., Dickinson A. (2020). New approaches for the treatment of chronic graft-versus-host disease: current status and future directions. Front Immunol.

[8] Wolff D., Greinix H., Lee S.J. (2018). Biomarkers in chronic graft-versus-host disease: quo vadis?. Bone Marrow Transplant.

[9] Milosevic E., Babic A., Iovino L., Markovic M., Grce M., Greinix H. (2022). Use of the NIH consensus criteria in cellular and soluble biomarker research in chronic graft-versus-host disease: a systematic review. Front Immunol.

[10] Peric Z., Schoemans H., Peczynski C. (2021). Management of steroid-refractory graft-versus-host disease - a survey by the transplant complications working party of the EBMT. Blood.

[11] Holtzman N.G., Pavletic S.Z. (2022). The clinical landscape of chronic graft-versus-host disease management in 2021. Br J Haematol.

[12] Sarantopoulos S., Cardones A.R., Sullivan K.M. (2019). How I treat refractory chronic graft-versus-host disease. Blood.

[13] Zeiser R., Sarantopoulos S., Blazar B.R. (2018). B-cell targeting in chronic graft-versus-host disease. Blood.

[14] Zeiser R., Blazar B.R. (2017). Pathophysiology of chronic graft-versus-host disease and therapeutic targets. N Engl J Med.

[15] Sarantopoulos S., Ritz J. (2015). Aberrant B-cell homeostasis in chronic GVHD. Blood.

[16] Allen J.L., Fore M.S., Wooten J. (2012). B cells from patients with chronic GVHD are activated and primed for survival via BAFF-mediated pathways. Blood.

[17] Jia W., Poe J.C., Su H. (2021). BAFF promotes heightened BCR responsiveness and manifestations of chronic GVHD after allogeneic stem cell transplantation. Blood.

[18] Li X., Gao Q., Feng Y., Zhang X. (2019). Developing role of B cells in the pathogenesis and treatment of chronic GVHD. Br J Haematol.

[19] Jia W., Poe J.C., Su H. (2016). Recipient-derived BAFF and alloantigen synergistically activate B cells in murine chronic Gvhd. Blood.

[20] Malard F., Labopin M., Yakoub-Agha I. (2017). Rituximab-based first-line treatment of cGVHD after allogeneic SCT: results of a phase 2 study. Blood.

[21] Solomon S.R., Sizemore C.A., Ridgeway M. (2019). Safety and efficacy of rituximab-based first line treatment of chronic GVHD. Bone Marrow Transplant.

[22] Peng Y., Chen X., Liu Q. (2014). Alteration of naïve and memory B-cell subset in chronic graft-versus-host disease patients after treatment with mesenchymal stromal cells. Stem Cells Transl Med.

[23] Xuan L., Wang Y., Huang F. (2020). Sorafenib maintenance in patients with FLT3-ITD acute myeloid leukaemia undergoing allogeneic haematopoietic stem-cell transplantation: an open-label, multicentre, randomised phase 3 trial. Lancet Oncol.

[24] Jagasia M.H., Greinix H.T., Arora M. (2015). National institutes of health consensus development project on criteria for clinical trials in chronic graft-versus-host disease: I. The 2014 diagnosis and staging working group report. Biol Blood Marrow Transplant.

[25] Filipovich A.H., Weisdorf D., Pavletic S. (2005). National Institutes of Health consensus development project on criteria for clinical trials in chronic graft-versus-host disease: I. Diagnosis and staging working group report. Biol Blood Marrow Transplant.

[26] Hao Y., Hao S., Andersen-Nissen E. (2021). Integrated analysis of multimodal single-cell data. Cell.

[27] Pedregosa F., Varoquaux G., Gramfort A. (2011). Scikit-learn: machine learning in Python. J Mach Learn Res.

[28] Avni B., Neiman D., Shaked E. (2022). Chronic graft versus host disease detected by tissue-specific cell-free DNA methylation biomarkers. Blood.

[29] McInnes L., Healy J., Melville J. (2018). Umap: uniform manifold approximation and projection for dimension reduction. arXiv.

[30] Traag V.A., Waltman L., Van Eck N.J. (2019). From Louvain to Leiden: guaranteeing well-connected communities. Sci Rep.

[31] Kitko C.L., Pidala J., Schoemans H.M. (2021). National institutes of Health consensus development project on criteria for clinical trials in chronic graft-versus-host disease: IIa. The 2020 clinical implementation and early diagnosis working group report. Transplant Cell Ther.

[32] Cuvelier G.D.E., Nemecek E.R., Wahlstrom J.T. (2019). Benefits and challenges with diagnosing chronic and late acute GVHD in children using the NIH consensus criteria. Blood.

[33] Carpenter P.A., Logan B.R., Lee S.J. (2018). A phase II/III randomized, multicenter trial of prednisone/sirolimus versus prednisone/sirolimus/calcineurin inhibitor for the treatment of chronic graft-versus-host disease: BMT CTN 0801. Haematologica.

[34] Pusic I., Pavletic S.Z. (2019). Challenges in conducting studies in chronic graft-versus-host disease. Clin Hematol Int.

[35] Schoemans H.M., Lee S.J., Ferrara J.L. (2018). EBMT−NIH−CIBMTR Task Force position statement on standardized terminology & guidance for graft-versus-host disease assessment. Bone Marrow Transplant.

[36] McManigle W., Youssef A., Sarantopoulos S. (2019). B cells in chronic graft-versus-host disease. Hum Immunol.

[37] Tsai C.Y., Oo M., Peh J.H. (2024). Splenic marginal zone B cells restrict Mycobacterium tuberculosis infection by shaping the cytokine pattern and cell-mediated immunity. Cell Rep.

[38] Yan S.X., Deng X.M., Wang Q.T., Sun X.J., Wei W. (2015). Prednisone treatment inhibits the differentiation of B lymphocytes into plasma cells in MRL/MpSlac-lpr mice. Acta Pharmacol Sin.

[39] Podgorny P.J., Liu Y., Dharmani-Khan P. (2014). Immune cell subset counts associated with graft-versus-host disease. Biol Blood Marrow Transplant.

[40] Misumi I., Whitmire J.K. (2014). B cell depletion curtails CD4+ T cell memory and reduces protection against disseminating virus infection. J Immunol.

[41] Dubovsky J.A., Flynn R., Du J. (2014). Ibrutinib treatment ameliorates murine chronic graft-versus-host disease. J Clin Invest.

[42] Flynn R., Du J., Veenstra R.G. (2014). Increased T follicular helper cells and germinal center B cells are required for cGVHD and bronchiolitis obliterans. Blood.

[43] Wang K.S., Kim H.T., Nikiforow S. (2017). Antibodies targeting surface membrane antigens in patients with chronic graft-versus-host disease. Blood.

[44] Martínez-Laperche C., Buces E., Aguilera-Morillo M.C. (2018). A novel predictive approach for GVHD after allogeneic SCT based on clinical variables and cytokine gene polymorphisms. Blood Adv.

[45] Greinix H.T., Kuzmina Z., Weigl R. (2015). CD19+CD21low B cells and CD4+CD45RA+CD31+ T cells correlate with first diagnosis of chronic graft-versus-host disease. Biol Blood Marrow Transplant.

[46] Cuvelier G.D.E., Ng B., Abdossamadi S. (2023). A diagnostic classifier for pediatric chronic graft-versus-host disease: results of the ABLE/PBMTC 1202 study. Blood Adv.

[47] Weissinger E.M., Human C., Metzger J. (2017). The proteome pattern cGvHD_MS14 allows early and accurate prediction of chronic GvHD after allogeneic stem cell transplantation. Leukemia.

[48] Khoder A., Alsuliman A., Basar R. (2017). Evidence for B cell exhaustion in chronic graft-versus-host disease. Front Immunol.

[49] Ji R., Li Y., Huang R., Xiong J., Wang X., Zhang X. (2023). Recent advances and research progress in biomarkers for chronic graft versus host disease. Crit Rev Oncol Hematol.

[50] Giesen N., Schwarzbich M.A., Dischinger K. (2020). CXCL9 predicts severity at the onset of chronic graft-versus-host disease. Transplantation.

[51] Levine J.E., Braun T.M., Harris A.C. (2015). A prognostic score for acute graft-versus-host disease based on biomarkers: a multicentre study. Lancet Haematol.

