# Supplementary material for: Dynamic forecasting module for chronic graft-versus-host disease progression based on a disease-associated subpopulation of B cells: a multicenter prospective study
Source: eBioMedicine. 2025 Feb 12;113:105587. doi: 10.1016/j.ebiom.2025.105587 (PMC11872411; doi:10.1016/j.ebiom.2025.105587)
Supplement: Supplementary Figures [file mmc1.docx]

**Figure S1. Gating strategy for classical B cell subsets.**

**
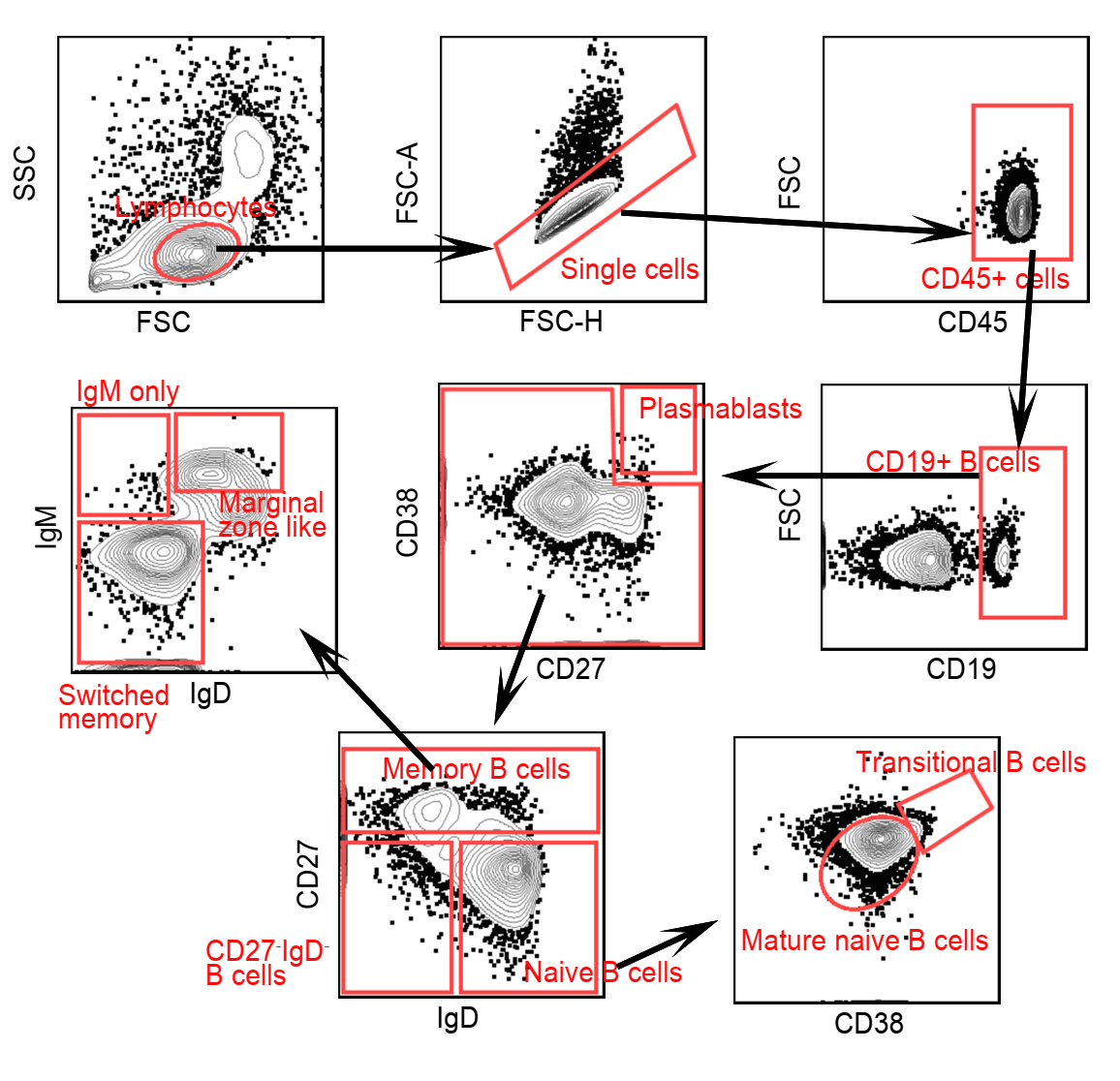
**

**Figure S2. Gating strategy for cGVHD related B cell subset.**

**
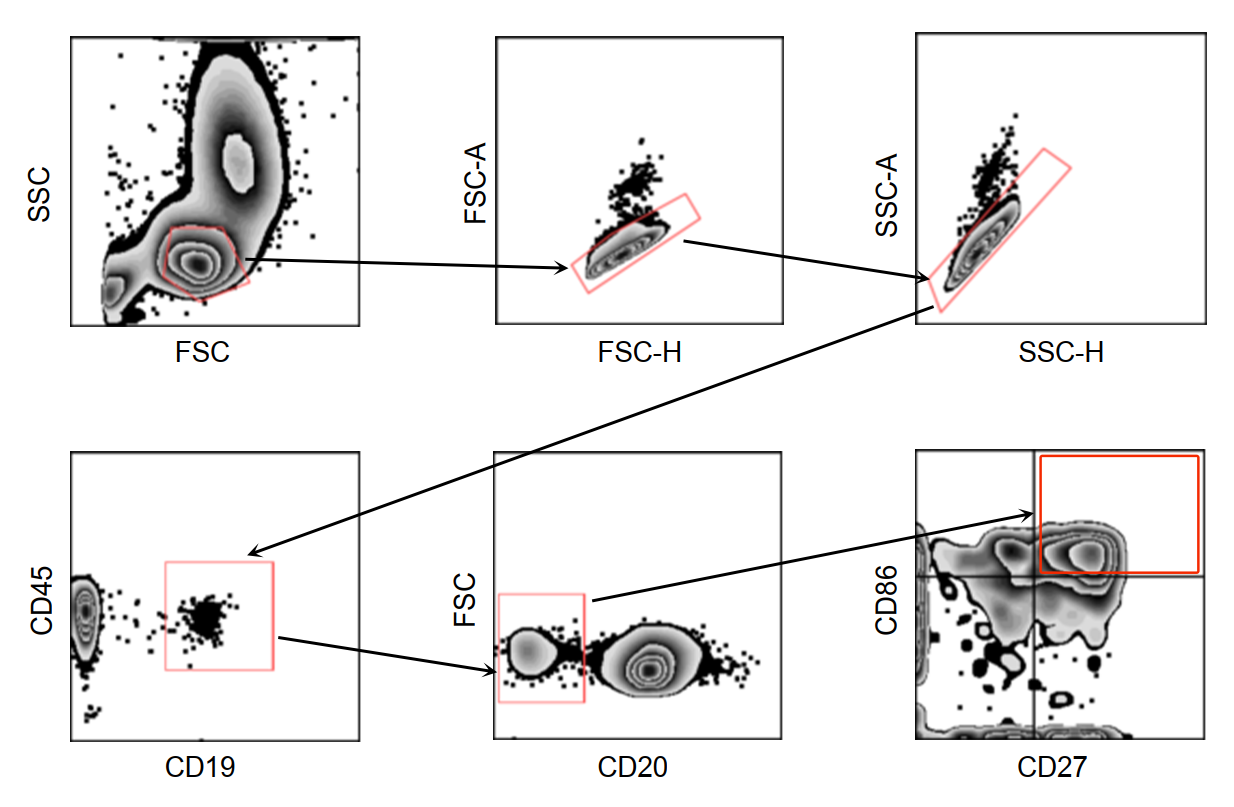
**

**Figure S3.  Trial profile**


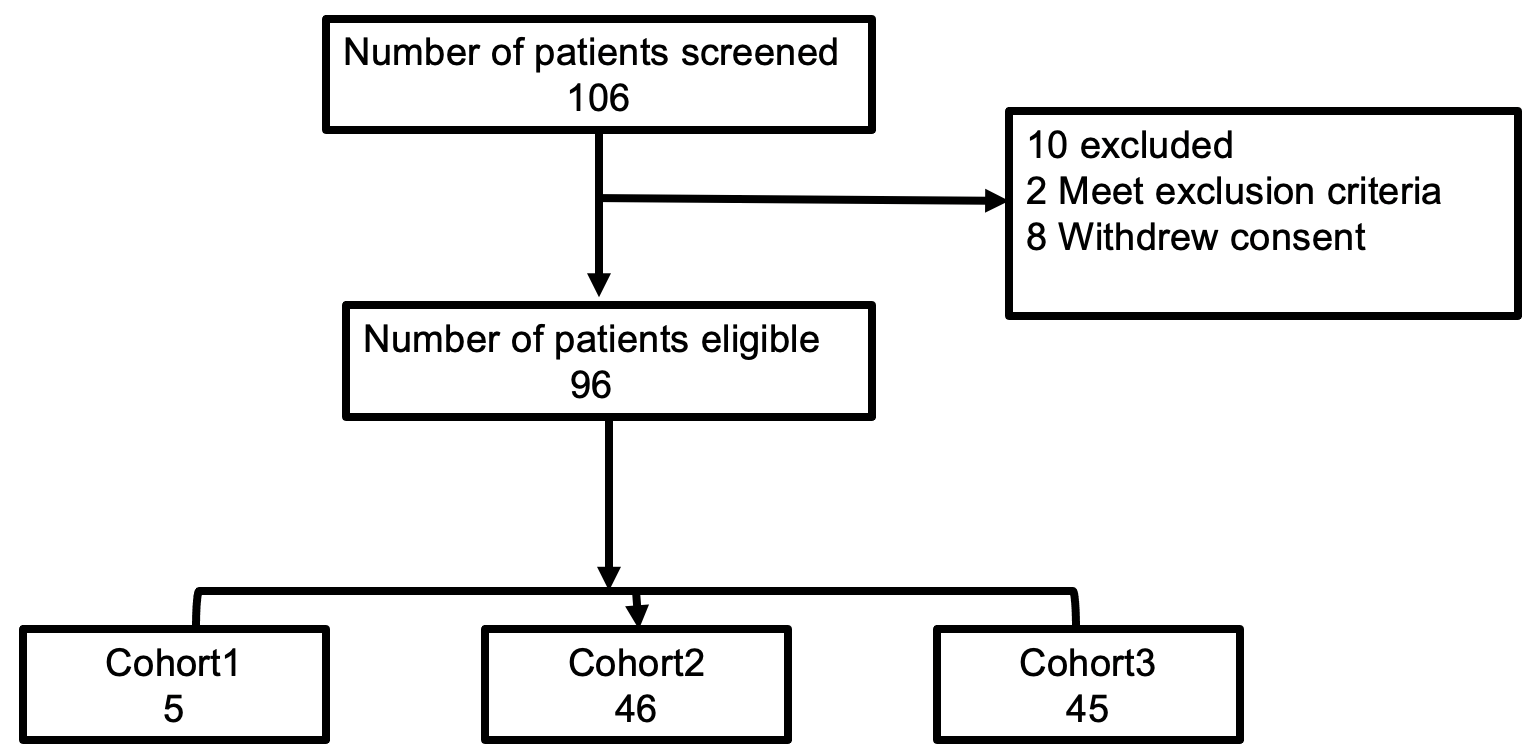


**Figure S4. Repeated experiment of cohort1 using non-GVHD compared with cGVHD patients.**

**
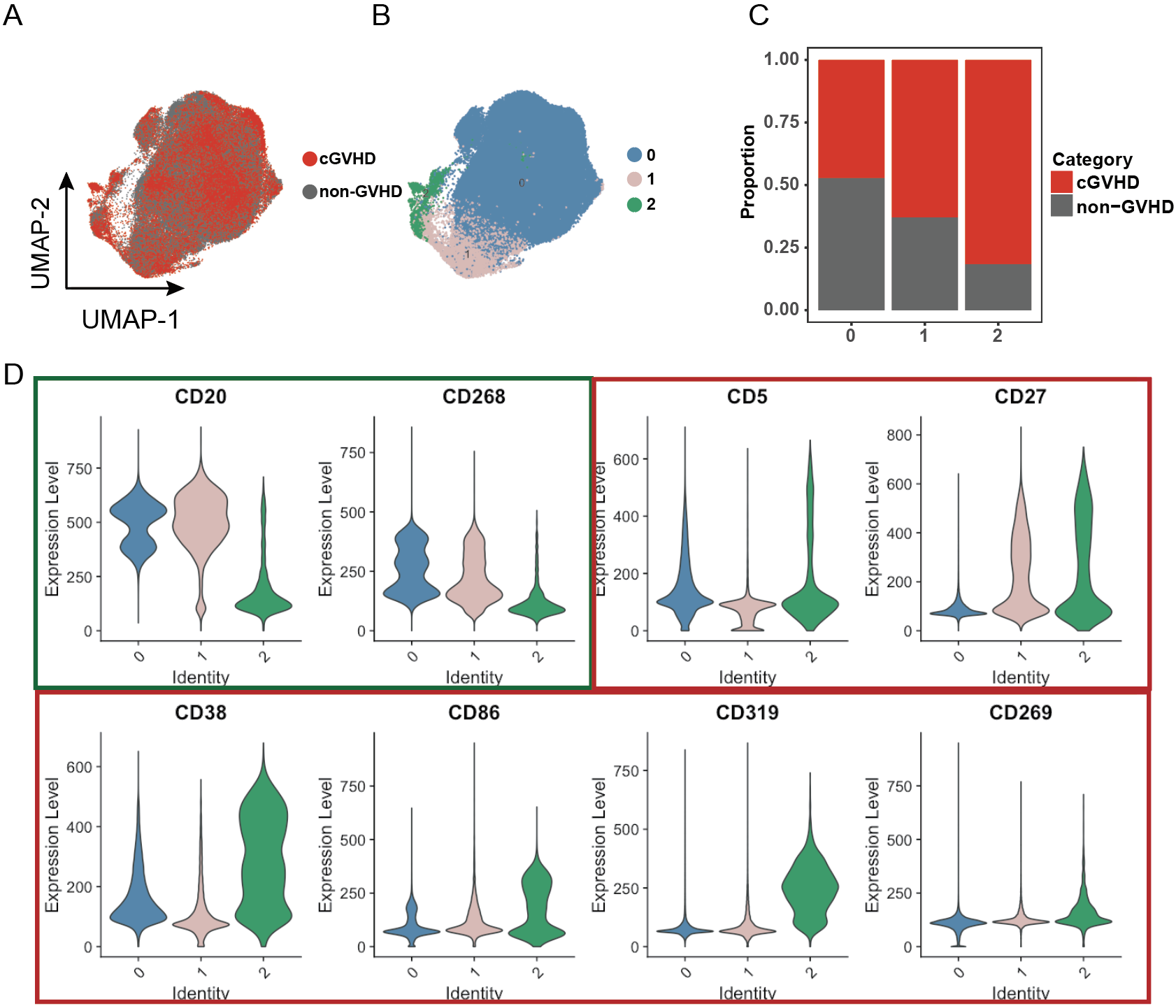
**

(A) UMAP plot for the B cell samples of 10 participants. Cell coloring indicates the sample source: red, cGVHD patients; gray, non-GVHD patients.

(B) UMAP plot for the B cell samples of 10 participants. Cells are colored to indicate participants category (left panel) and cell cluster(right panel).

(C) Bar plot of cell proportions (y-axis) contained in each Louvain cell cluster (x-axis). Bar color indicates the sample source: red, cGVHD patients; gray, non-GVHD patients. Bar length represents the percentage of the sample under the corresponding cluster.

(D) Violin plot of the markers selected as potentially defining cGVHD-related cell clusters. The x-axis in each plot represents the Louvain cell clusters, while the y-axis represents the relative expression level of each marker. The width of each curve corresponds to the approximate frequency of data.

**Figure S5. cGPS values comparison between healthy donors and cGVHD patients within cohort3**

**
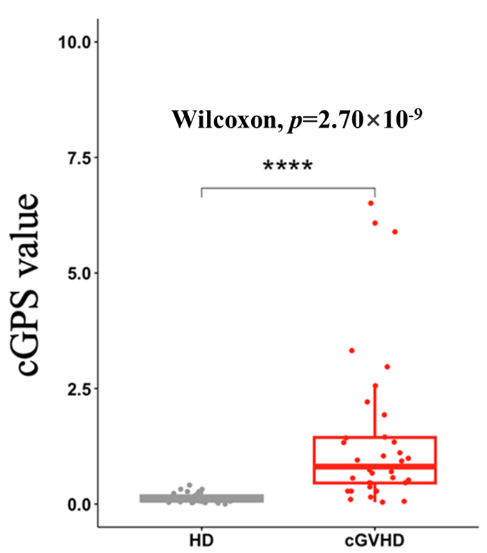
**

Statistical analysis of differences in the cGPS among cGVHD and healthy donor samples. The x-axis represents the healthy donors (HD, gray) and cGVHD patients (red). The y-axis presents the corresponding cGPS score. The Wilcoxon represents Wilcoxon rank sum test.

**Figure S6. The time data for all cGVHD patients.**

**
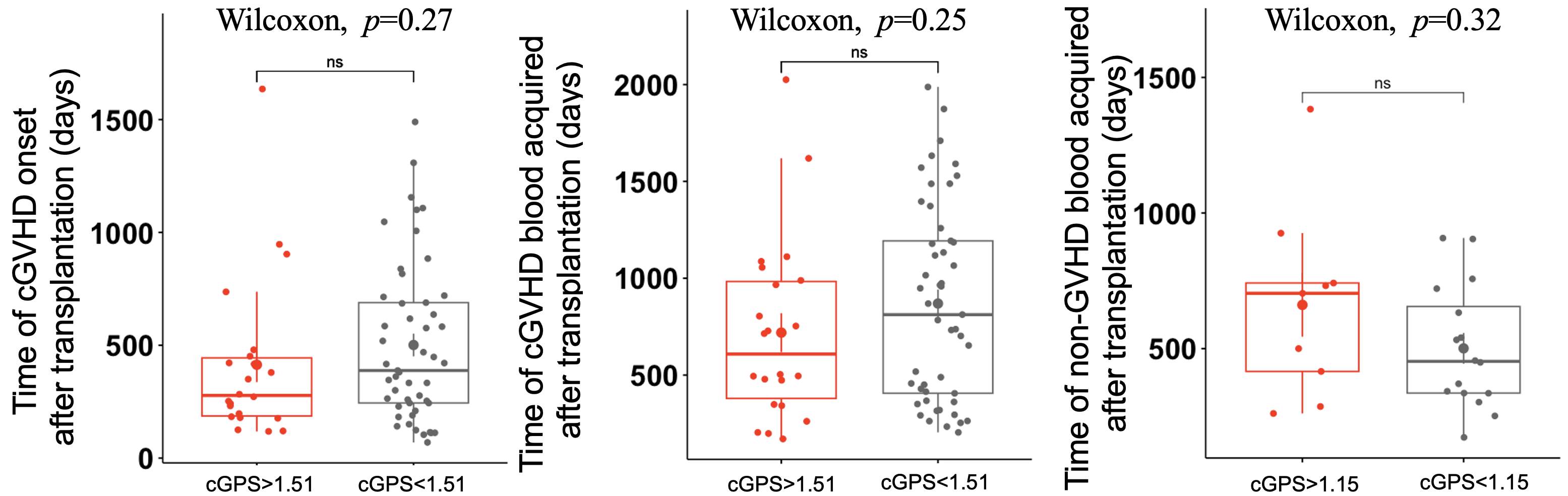
**

The dot on the box represents each patient. The y axis represents the time of cGVHD onset after transplantation (left), blood acquired after transplantation (middle), non-GVHD blood acquired after transplantation (right). The color of box represents cGPS category, for cGVHD group: cGPS>1.51 (red) and cGPS<1.51(grey), for non-GVHD group: cGPS>1.15 (red) and cGPS<1.15 (grey). Wilcoxon rank sum test was conducted, ns represents no significance.
